# Nicorandil Improves Left Ventricular Myocardial Strain in Patients With Coronary Chronic Total Occlusion

**DOI:** 10.3389/fcvm.2022.864223

**Published:** 2022-05-12

**Authors:** Shaomin Chen, Chen Ma, Xinheng Feng, Ming Cui

**Affiliations:** ^1^Department of Cardiology and Institute of Vascular Medicine, Peking University Third Hospital; Key Laboratory of Cardiovascular Molecular Biology and Regulatory Peptides, Ministry of Health; Key Laboratory of Molecular Cardiovascular Science, Ministry of Education, Beijing Key Laboratory of Cardiovascular Receptors Research, Beijing, China; ^2^Healthcare Department, National Center of Gerontology, Beijing Hospital, Beijing, China; ^3^Institute of Geriatric Medicine, Chinese Academy of Medical Sciences, Beijing, China

**Keywords:** chronic total occlus, nicorandil, speckle tracking echocardiograph, global area strain, coronary heart disease

## Abstract

**Background:**

Nicorandil is recommended as a second-line treatment for stable angina; however, randomized-controlled trials to evaluate the benefit of nicorandil for patients with chronic total occlusion (CTO) are lacking.

**Objective:**

To determine whether nicorandil can improve left ventricular (LV) myocardial strain in patients with CTO.

**Methods:**

Patients with CTO were included and randomized to the nicorandil group (*n* = 31) and the control group (*n* = 30). Nicorandil was given orally at 15 mg/day for 3 months in the nicorandil group. Three-dimensional speckle-tracking echocardiography and the Seattle Angina Questionnaire (SAQ) survey were performed at baseline and at 3 months. The primary study endpoint was the LV global area strain (GAS) at 3 months.

**Results:**

The nicorandil and the control groups were well-matched at baseline, including the mean GAS and SAQ scores. At 3 months, GAS in the nicorandil group was significantly higher than that in the control group (−23.7 ± 6.3% vs. −20.3 ± 5.6%, respectively; *p* = 0.033). There were no significant differences in LV global longitudinal strain, global circumferential strain, global radial strain, LV ejection fraction, LV end-diastolic volume, and LV end-systolic volume at 3 months between the two groups. At 3 months, the SAQ scores for angina stability, angina frequency, and treatment satisfaction in the nicorandil group were significantly higher than those in the control group.

**Conclusion:**

Nicorandil treatment can improve GAS and angina symptoms in patients with CTO.

**Clinical Trial Registration:**

www.ClinicalTrials.gov, identifier: NCT05087797.

## Introduction

Coronary chronic total occlusion (CTO) refers to a coronary artery with a complete obstruction resulting in thrombolysis in myocardial infarction grade 0 flows for over 3 months (1). The prevalence of CTO ranges between 30% and 50% among patients with coronary artery disease (CAD) (1). Although collaterals are capable of maintaining myocardial viability, they are insufficient to sustain myocardial perfusion during exercise, which can lead to angina (2). Furthermore, CTO is associated with a higher mortality rate (3, 4).

Observational studies and registries showed that successful CTO percutaneous coronary intervention (PCI) or coronary artery bypass grafting (CABG) was associated with improvements in angina pectoris symptoms, left ventricular (LV) function, quality of life, and survival (5–8). However, randomized-controlled trials (RCTs) failed to show improvements in LV function and prognosis compared with optimal medical therapy (OMT) (6). Moreover, the success rate in CTO PCI was lower than that in non-CTO PCI, and the complication rate was higher than that in non-CTO PCI (9). OMT remains the cornerstone for the management of patients with CTO. There are few studies focusing on medical therapy in patients with CTO and stable angina. Therefore, these patients are usually managed with medical therapy for stable angina. To prevent angina and ischemia, beta-blockers, calcium channel blockers, and nitrates are the primary agents (10). Nicorandil, an adenosine triphosphate (ATP)-sensitive potassium ATP channel opener with nitrate-like effects, is recommended as a second-line treatment for chronic coronary syndrome by The European Society of Cardiology (10). However, RCTs to evaluate the benefit of nicorandil for patients with CTO are lacking. In the past decade, deformation imaging has developed rapidly, and three-dimensional-speckle tracking echocardiography (3D-STE) is becoming a valuable method in the comprehensive assessment of myocardial function (11). 3D-STE can detect subclinical LV dysfunction using strain parameters in CAD patients without LV regional wall motion abnormality (11). Strain parameters derived from 3D-STE comprise global longitudinal strain (GLS), global circumferential strain (GCS), global radial strain (GRS), and global area strain (GAS). Among these parameters, GAS is ideal to detect severe coronary stenosis (12, 13). This study aimed to determine whether nicorandil can improve LV myocardial strain in patients with CTO.

## Materials and Methods

### Patients

This was a single-center, open-label, RCT performed at Peking University Third Hospital, and the trial was registered at https://clinicaltrials.gov (NCT05087797). Patients with CTO identified on coronary angiography between January 2018 and November 2019 were screened for inclusion in this study. The inclusion criteria were: age between 18 and 80 years and one or more CTO vessels (≥ 2 mm in diameter). The exclusion criteria were: successful PCI for all CTO lesions; ST segment elevation myocardial infarction during the past 3 months; planned CABG within 3 months; valvular heart disease or congenital heart disease; cardiomyopathy; heart failure with New York Heart Association class ≥ III; persistent atrial fibrillation or complete left bundle branch block; and severe liver, kidney, or lung disease. Participants were randomized according to an IBM SPSS version 23.0 (IBM Corp., Armonk, NY, USA)-generated randomization schedule, with a 1:1 allocation to the nicorandil group or the control group. All patients in this study received standard treatment for CAD, and patients in the nicorandil group were also given nicorandil (Tohoku Nipro Pharmaceutical Corporation, Tokyo, Japan) 5 mg orally thrice per day for 3 months.

### Coronary Angiographic Assessment

Chronic total occlusion was defined as coronary artery occlusion for over 3 months. The duration was estimated on the basis of a history of an ischemic event potentially related to the coronary occlusion, or previous coronary angiography confirming the occluded vessel. Non-CTO PCI was performed, if needed. Coronary angiograms were reviewed by one specialist who was blinded to the group allocation. Collateral channels and their Rentrop classification were analyzed.

### 3D Echocardiography and Speckle-Tracking Analysis

All patients underwent 3D echocardiography before randomization (baseline) and 3 months after treatment, with a Vivid 95 (GE Healthcare, Wauwatosa, WI, USA) system and a 4 V-D transducer (GE Healthcare). Patients were placed in the left lateral position, and 3D images of the LV were obtained from the apical window. Multibeat-3D model was used to obtain the full-volume dataset from four consecutive cardiac cycles. The frame rate was ≥ 25 Hz to perform the speckle-tracking analysis. Images were recorded digitally and analyzed offline using the 4D Auto LVQ package (EchoPAC V.110.1.3; GE Healthcare). The LV endocardial border was detected automatically and was adjusted manually, if needed. LV end-diastolic volume (LVEDV) and LV end-systolic volume (LVESV) were calculated, and 3D LV ejection fraction (LVEF) was provided. Subsequently, automatic tracing of the epicardial border was performed to identify the region of interest required for speckle-tracking analysis. The epicardial tracing was also manually adjusted to include the entire LV wall. Next, the strain parameters were displayed as the GLS, GCS, GRS, and GAS of the LV. Echocardiographic analyses were performed by a single experienced cardiologist who was blinded to the patients' clinical and study data.

Intra- and inter-observer reproducibility for the 3D echocardiographic parameters were assessed in 20 randomly selected patients. To test intra-observer reproducibility, echocardiographic analyses were performed twice by the same cardiologist 1 week apart. To test inter-observer reproducibility, echocardiographic analyses were performed again by a second cardiologist.

### Seattle Angina Questionnaire

The 19-item SAQ survey was conducted at baseline and again at 3 months. This questionnaire measures health status across five domains: physical limitation, angina stability, angina frequency, treatment satisfaction, and quality of life. All domain scores range from 0 to 100, with higher scores indicating fewer symptoms and better health status (14).

### Study Endpoints

The primary study endpoint was GAS at 3 months. The secondary study endpoints were GLS, GCS, GRS, LVEF, LVEDV, and LVESV at 3 months, and health status measured by SAQ at 3 months.

### Sample Size Estimation and Statistical Analysis

According to previous studies, GAS for the nicorandil group and the control group were estimated to be −18 ± 4% and −15 ± 4%, respectively (12, 13). Therefore, in this study, each group required 31 samples for an alpha level of 0.05 and 80% power using a two-sided test, allowing for 15% attrition.

SPSS 23.0 (IBM Corp.) was used for the statistical analysis. The Kolmogorov–Smirnov method was used to evaluate the normality of the continuous data. Continuous variables with normal distribution were expressed as mean ± standard deviation, and comparisons between the two groups were performed using Student's *t*-test. Continuous variables with non-normal distribution were expressed as median (25th, 75th percentiles), and comparisons between the two groups were performed using the Mann–Whitney *U* test. Categorical variables were expressed as numbers (percentages), and comparisons between the two groups were performed using the Chi-square test. The intra-group correlation coefficient was used to test the reproducibility of the 3D echocardiographic parameters between examiners and within examiners. An intra-group correlation coefficient > 0.75 indicated good repeatability. Pearson correlation was used to identify the correlations between strain parameters and SAQ scores.

## Results

### Study Groups

**Figure 1:**
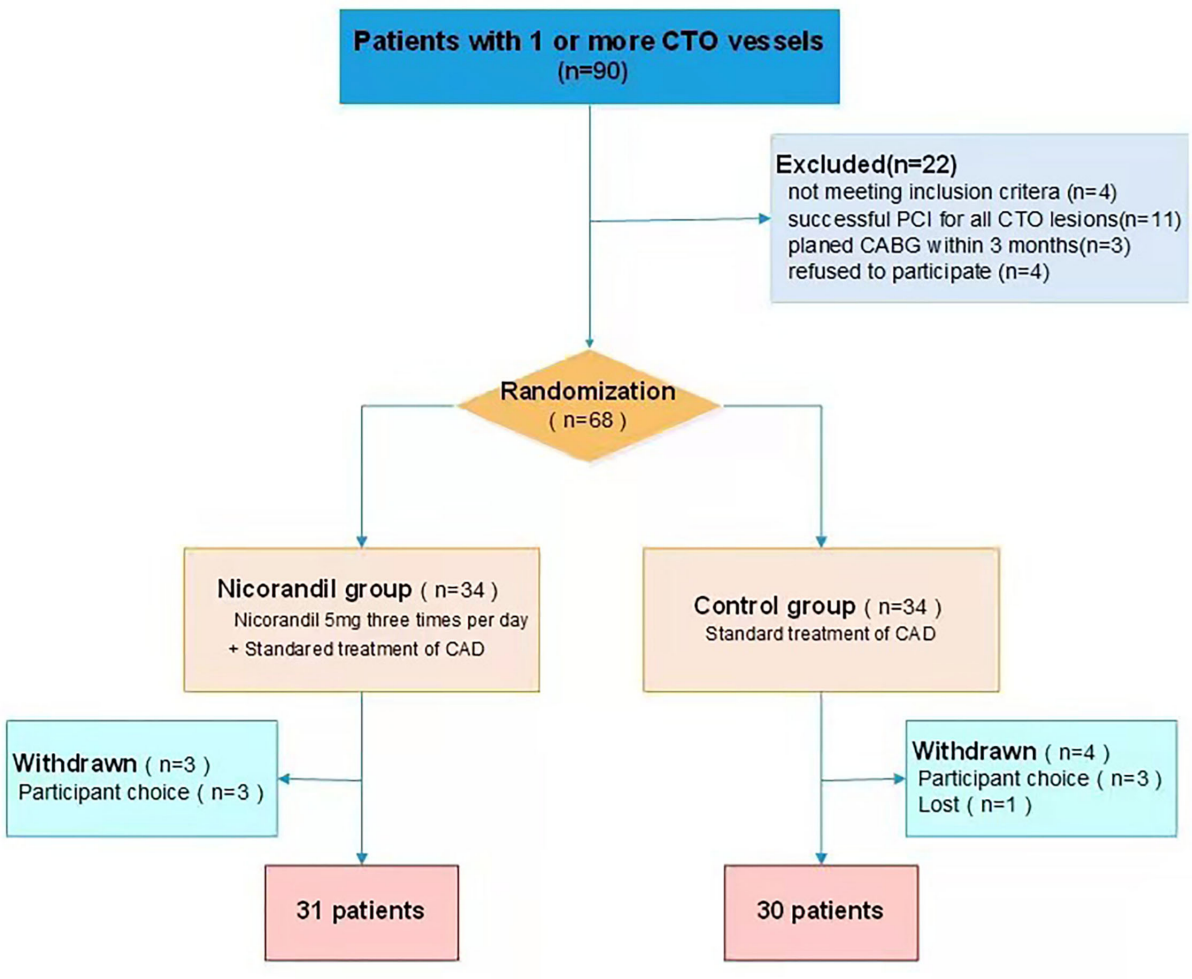
Patient flow chart. CABG, coronary artery bypass grafting; CTO, chronic total occlusion; PCI, percutaneous coronary intervention.

During the study period, 90 consecutive patients with CTO were screened for participation. Of all eligible patients, 68 patients were randomized to the nicorandil group or the control group. Seven patients withdrew after randomization. Data for a final 61 patients (31 patients in the treatment group and 30 patients in the control group) were analyzed (Figure 1). The mean age was 62.8 ± 9.6 years, and 49 (80.3%) patients were men. Most patients were on aspirin, P2Y12 receptor antagonist, and statin treatment. A total of 37 (60.7%) patients were given β-blockers, 26 (42.6%) were given nitrates, 24 (39.3%) were given calcium-channel blockers, and 5 (8.2%) were given ivabradine. Participants in the treatment group and the control group were well-matched in terms of their clinical characteristics (Table 1).

**Table 1:** Demographic and clinical characteristics of the nicorandil group and the control group.

	**Total (*n* = 61)**	**Control group (*n* = 30)**	**Nicorandil group (*n* = 31)**	***p***
Age, years	62.8 ± 9.6	62.2 ± 11.0	63.5 ± 8.3	0.608
Male, *n* (%)	49 (80.3)	25 (83.3)	24 (77.4)	0.561
BMI, kg/m^2^	25.8 ± 3.1	26.1 ± 2.9	25.6 ± 3.4	0.593
Heart rate, beats/min	74.5 ± 10.2	75.8 ± 10.8	73.3 ± 9.7	0.344
Systolic blood pressure, mmHg	135.8 ± 19.5	139.0 ± 22.7	132.7 ± 15.6	0.211
Hypertension, *n* (%)	45 (73.8)	21 (70.0)	24 (77.4)	0.510
Diabetes mellitus, *n* (%)	34 (55.7)	19 (63.3)	15 (48.4)	0.240
Dyslipidemia, *n* (%)	38 (62.3)	16 (53.3)	22 (71.0)	0.150
Current smoker, *n* (%)	35 (57.4)	17 (56.7)	18 (58.1)	0.912
Previous myocardial infarction, *n* (%)	26 (42.6)	15 (50.0)	11 (35.5)	0.252
Previous stroke, *n* (%)	8 (13.1)	3 (10.0)	5 (16.1)	0.707
Peripheral artery disease, *n* (%)	17 (27.9)	8 (26.7)	9 (29.0)	0.837
Previous PCI, *n* (%)	20 (32.8)	7 (23.3)	13 (41.9)	0.122
Previous CABG, *n* (%)	5 (8.2)	1 (3.3)	4 (12.9)	0.354
Diagnosis				0.753
SCAD, *n* (%)	27 (44.3)	15 (50)	12 (38.7)	
UAP, *n* (%)	27 (44.3)	10 (33.3)	17 (54.8)	
NSTEMI, *n* (%)	7 (11.5)	5 (16.7)	2 (6.5)	
HGB (g/L)	138.3 ± 14.3	139.1 ± 13.2	137.5 ± 15.4	0.656
Scr (umol/L)	85.6 ± 16.2	84.7 ± 13.3	86.5 ± 18.7	0.670
TG (mmol/L)	1.6 ± 0.7	1.7 ± 0.9	1.5 ± 0.6	0.262
TC (mmol/L)	4.0 ± 0.9	4.2 ± 0.8	3.8 ± 0.9	0.097
HDL-C (mmol/L)	1.1 ± 0.2	1.1 ± 0.3	1.0 ± 0.2	0.172
LDL-C (mmol/L)	2.4 ± 0.9	2.6 ± 0.8	2.2 ± 0.9	0.075
Glu (mmol/L)	6.3 (5.3, 8.1)	6.3 (5.3, 8.7)	6.2 (5.3, 7.2)	0.535
hs-CRP (mg/L)	1.7 (0.6, 5.0)	1.9 (0.8, 5.6)	1.1 (0.3, 3.2)	0.098
NT-proBNP (pg/ml)	132.7 (62.0, 272.8)	188.1 (81.9, 360.2)	105.8 (56.3, 216.5)	0.076
Medications				
Aspirin, *n* (%)	57 (93.4)	29(96.7)	28(90.3)	0.317
P2Y_12_ receptor antagonist, *n* (%)	53 (86.9)	25 (83.3)	28 (90.3)	0.419
Statin, *n* (%)	61 (100)	30 (100)	31 (100)	-
β blocker, *n* (%)	37 (60.7)	16 (53.3)	21 (67.7)	0.249
ACEi/ARB, *n* (%)	35 (57.4)	20 (66.7)	15 (48.4)	0.149
Nitrates, *n* (%)	26 (42.6)	11 (42.3)	15 (48.4)	0.355
CCB, *n* (%)	24 (39.3)	10 (33.3)	14 (45.2)	0.344
Ivabradine, *n* (%)	5 (8.2)	2 (6.7)	3 (9.7)	0.639

*Data are expressed as a mean ± standard deviation, median (25th, 75th percentiles), or number (percentage). ACEi, ACE inhibitor; ARB, angiotensin receptor blocker; BMI, body mass index; CABG, coronary artery bypass grafting; CCB, calcium-channel blocker; Glu, glucose; HDL-C, High density liptein cholesterol; HGB, hemoglobin; hsCRP, high-sensitivity C-reaction protein; LAD, left anterior descending artery; LCX, left circumflex artery; LDL-C, low-density liptein cholesterol; NT-proBNP, N-terminal pro-B-type natriuretic peptide; NSTEMI, non-ST-segment elevation myocardial infarction; PCI, percutaneous coronary intervention; RCA, right coronary artery; SCAD, stable coronary artery disease; Scr, serum creatinine; TC, total cholesterol; TG, triglyceride; UAP, unstable angina pectoris*.

### Coronary Angiography and Procedural Data

Among the patients included in this study, 53 (86.9%) patients had a single CTO vessel, and 8 (13.1%) patients had two CTO vessels. The right coronary artery was the most common location of occlusions, followed by the left circumflex artery and left anterior descending artery. CTO PCI was attempted in 23 (37.7%) patients. A total of 38 (62.3%) patients had Rentrop 2 or Rentrop 3 collateral channels, and 32 (52.5%) patients underwent non-CTO PCI. Coronary angiography and procedural data were comparable between the treatment group and the control group (Table 2).

**Table 2:** Baseline angiographic and procedural characteristics of the nicorandil group and the control group.

	**Total (*n* = 61)**	**Control group (*n* = 30)**	**Nicarandil group (*n* = 31)**	***p***
Numbers of diseased vessels, *n* (%)				0.836
1	9 (14.8)	4 (13.3)	5 (16.1)	
2	20 (32.8)	10 (33.3)	10 (32.3)	
3	32 (52.5)	16 (53.3)	16 (51.6)	
Numbers of CTO vessels, *n* (%)				0.478
1	53 (86.9)	27 (50.9)	26 (49.1)	
2	8 (13.1)	3 (37.5)	5 (62.5)	
CTO distribution				
LAD, *n*%	12 (19.7)	6 (20.0)	6 (19.4)	0.949
LCX, *n*%	23 (37.7)	13 (43.3)	10 (32.3)	0.372
RCA, *n*%	34 (55.7)	14 (46.7)	20 (64.5)	0.161
Attempted CTO-PCI, *n* (%)	23 (37.7)	11 (36.7)	13 (41.9)	0.674
Rentrop classification of collaterals, *n* (%)				0.222
0/1	23 (37.7)	9 (30.0)	14 (45.2)	
2/3	38 (62.3)	21 (70.0)	17 (54.8)	
Non-CTO PCI, *n* (%)	32 (52.5)	18 (60.0)	14 (45.2)	0.246

*Data are expressed as numbers (percentages). CTO, chronic total occlusion; LAD, left anterior descending; LCX, circumflex; RCA, right coronary artery; PCI, percutaneous coronary intervention*.

### Echocardiographic Data

**Figure 2:**
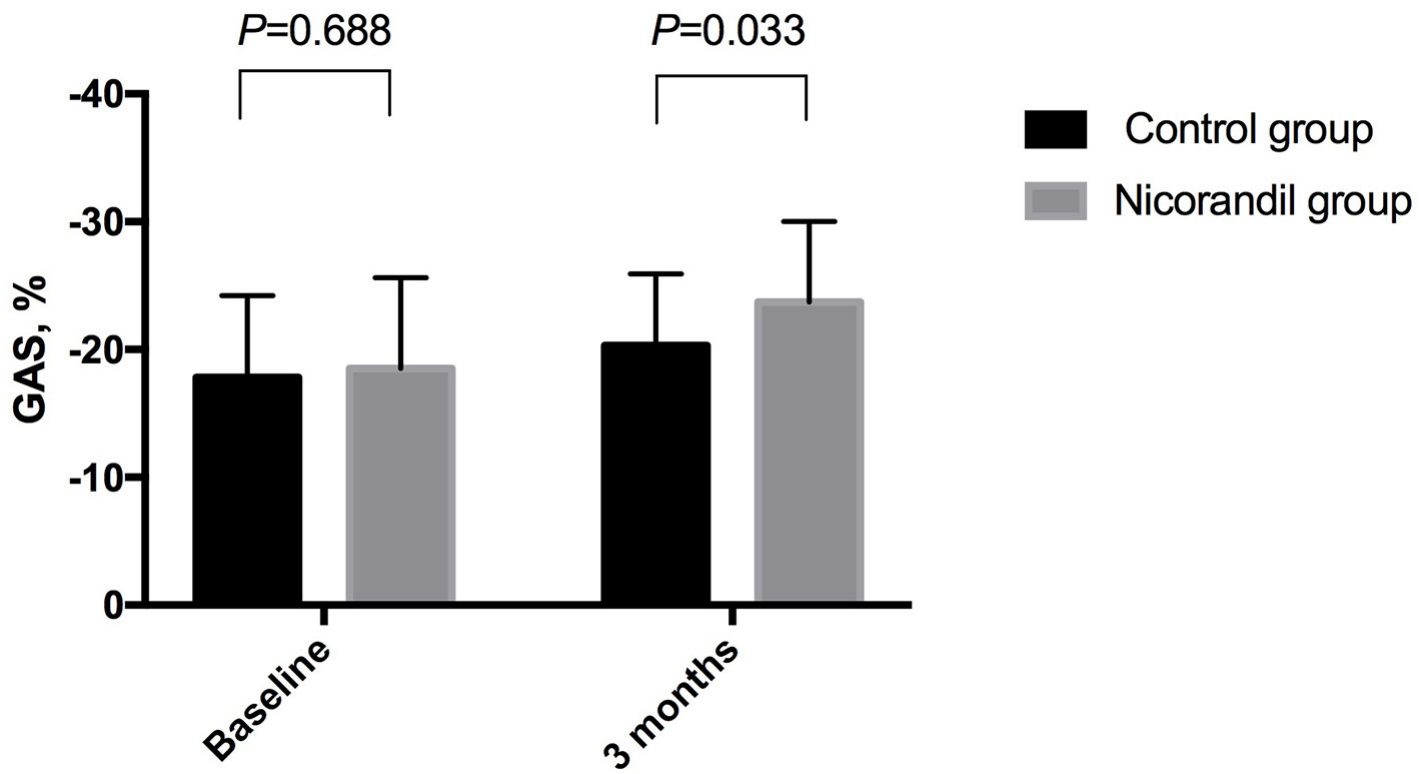
Comparison of GAS between the nicorandil group and the control group. GAS, global area strain.

There were no significant differences in baseline echocardiographic data between the two groups. At 3 months, GAS in the nicorandil group was significantly higher than that in the control group (−23.7 ± 6.3% vs. −20.3 ± 5.6%, respectively; *p* = 0.033; Table 3, Figure 2). There were no significant differences in GLS, GCS, GRS, LVEF, LVEDV, and LVESV at 3 months between the two groups (Table 3). The intra- and inter-observer reproducibility for the 3D echocardiographic parameters were good (Table 4).

**Table 3:** 3D echocardiographic data of the nicorandil group and the control group.

	**Baseline**			**3 months**		
	**Control group (*n* = 30)**	**Nicarandil group (*n* = 31)**	***P***	**Control group (*n* = 30)**	**Nicarandil group (*n* = 31)**	***p***
GAS, %	−17.8 ± 6.4	−18.5 ± 7.1	0.688	−20.3 ± 5.6	−23.7 ± 6.3	0.033
GLS, %	−9.8 ± 3.6	−9.6 ± 4.4	0.847	−11.2 ± 3.9	−12.3 ± 3.8	0.269
GCS, %	−10.7 ± 3.7	−11.4 ± 4.4	0.505	−12.9 ± 5.5	−12.3 ± 4.2	0.633
GRS, %	26.2 ± 9.9	25.6 ± 15.2	0.856	28.1 ± 7.7	29.5 ± 9.6	0.533
LVEF, %	55.2 ± 7.2	54.7 ± 6.9	0.783	56.8 ± 7.9	55.3 ± 7.8	0.459
LVEDV, ml	106.4 ± 19.6	104.3 ± 17.7	0.662	106.2 ± 18.8	104.9 ± 18.1	0.784
LVESV, ml	49.1 ± 12.6	48.2 ± 12.3	0.799	48.5 ± 11.0	47.1 ± 12.6	0.646

*Data are expressed as a mean ± standard deviation. GAS, global area strain; GCS, global circumferential strain; GLS, global longitudinal strain; GRS, global radial strain; LVEDV, left ventricular end-diastolic volume; LVEF, left ventricular ejection fraction; LVESV, left ventricular end-systolic volume*.

**Table 4:** Intra-observer and inter-observer reproducibility for 3D-STE parameters.

	**Intra-observer**	**Inter-observer**
GAS	0.932 (0.838–0.973)	0.942 (0.860–0.977)
GLS	0.872 (0.691–0.949)	0.891 (0.742–0.956)
GCS	0.865 (0.696–0.944)	0.907 (0.784–0.962)
GRS	0.961 (0.906–0.984)	0.963 (0.911–0.985)

*Data are expressed as intraclass correlation coefficient (ICC) with 95% confidence interval (CI). GAS, global area strain; GCS, global circumferential strain; GLS, global longitudinal strain; GRS, global radial strain*.

### SAQ Scores

**Figure 3:**
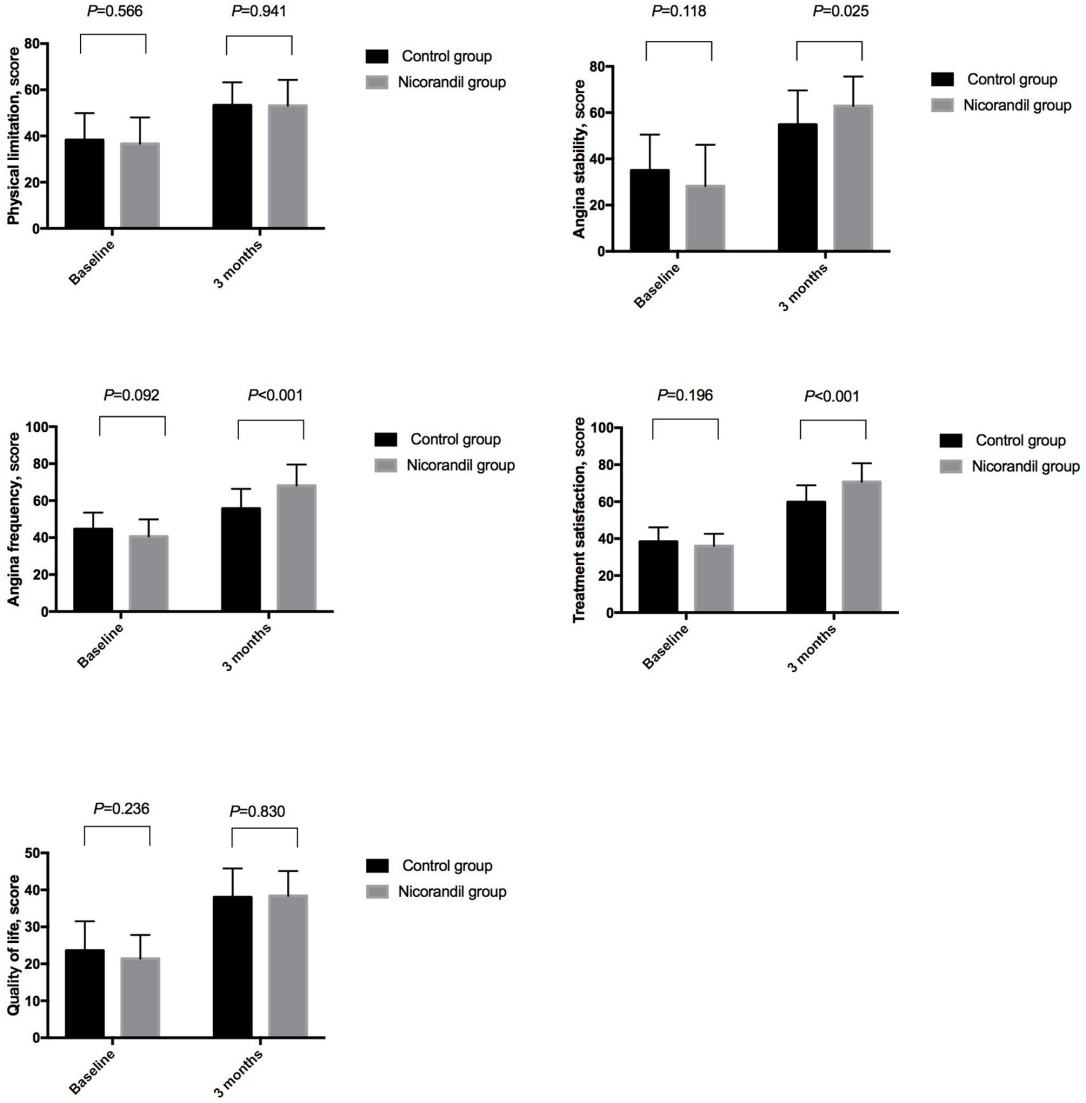
Comparison of seattle angina questionnaire scores between the nicorandil group and the control group.

Seattle angina questionnaire scores for all five domains were not significantly different between the nicorandil group and the control group at baseline. At 3 months, SAQ scores for angina stability, angina frequency, and treatment satisfaction were significantly higher in the nicorandil group than in the control group, while the scores for physical limitation and quality of life were not significantly different between the two groups (Table 5, Figure 3).

**Table 5:** Seattle angina questionnaire scores of the nicorandil group and the control group.

	**Baseline**			**3 months**		
	**Control group (*n* = 30)**	**Nicarandil group (*n* = 31)**	***p***	**Control group (*n* = 30)**	**Nicarandil group(*n* = 31)**	***p***
Physical limitation	38.3 ± 11.6	36.6 ± 11.4	0.566	53.3 ± 9.9	53.1 ± 11.2	0.941
Angina stability	35.0 ± 15.5	28.2 ± 17.9	0.118	54.8 ± 14.8	62.9 ± 12.7	0.025
Angina frequency	44.6 ± 8.9	40.6 ± 9.3	0.092	55.7 ± 10.7	68.1 ± 11.4	<0.001
Treatment satisfaction	38.4 ± 7.7	36.0 ± 6.6	0.196	59.8 ± 9.1	70.7 ± 10.1	<0.001
Quality of life	23.6 ± 7.9	21.4 ± 6.4	0.236	38.0 ± 7.8	38.4 ± 6.7	0.830

*Data are expressed as a mean ± standard deviation*.

### Relationship Between Strain Parameters and SAQ Scores

The absolute value of GAS was positively correlated with angina stability, angina frequency, and treatment satisfaction both at baseline and at 3 months. The absolute value of GLS was also positively correlated with angina stability, angina frequency, and treatment satisfaction both at baseline and at 3 months. However, GAS and GLS were not significantly correlated with a physical limitation or quality of life. GCS and GRS showed no significant relationship with SAQ scores (Table 6).

**Table 6:** Relationship of strain parameters with seattle angina questionnaire scores.

	**Physical limitation**		**Angina stability**		**Angina frequency**		**Treatment satisfaction**		**Quality of life**	
	***r***	***P***	***r***	***P***	***r***	***P***	***r***	***P***	***r***	***P***
Absolute value of GAS										
Baseline	0.246	0.056	0.546	<0.001	0.487	<0.001	0.404	0.001	0.159	0.225
3 months	0.002	0.988	0.514	<0.001	0.577	<0.001	0.348	0.006	0.194	0.138
Absolute value of GLS										
Baseline	0.123	0.347	0.385	0.002	0.504	<0.001	0.247	0.039	0.057	0.663
3 months	0.113	0.389	0.406	0.001	0.448	<0.001	0.284	0.027	0.170	0.194
Absolute value of GCS										
Baseline	0.090	0.496	0.230	0.078	0.229	0.078	0.056	0.672	0.005	0.967
3 months	−0.036	0.783	0.214	0.100	0.236	0.069	0.052	0.695	−0.073	0.580
GRS										
Baseline	0.069	0.598	0.222	0.085	0.202	0.119	0.125	0.336	−0.055	0.676
3 months	0.034	0.799	0.238	0.065	0.217	0.093	0.009	0.946	0.117	0.373

*GAS, global area strain; GCS, global circumferential strain; GLS, global longitudinal strain; GRS, global radial strain*.

## Discussion

This study has shown that nicorandil treatment as an adjunct to standard therapy improved GAS in patients with CTO. Nicorandil treatment also improved angina symptoms, as assessed by the SAQ. This study discusses their integration [(i.e., the key findings)] into the current understanding of the problem and how this advances and the current views speculate on the future direction of the research and freely postulate theories that could be tested in the future.

The potential benefits of CTO revascularization are still controversial. Although a number of observational studies have suggested that successful CTO PCI or CABG is associated with better cardiac function and long-term clinical outcomes (5–8), RCTs comparing CTO PCI plus OMT vs. OMT alone failed to show a reduction in clinical events or an improvement in LV function by CTO PCI (15–18). Thus, medications are fundamental in these patients.

Like nitrates, nicorandil dilates epicardial coronary arteries and increases coronary collateral blood flow *via* nitric oxide-mediated signaling pathways (19). In addition, nicorandil dilates the microvasculature to improve cardiac perfusion by activating potassium ATP channels (19). Nicorandil also protects cardiomyocytes by opening the mitochondrial potassium ATP channels in ischemia-reperfusion conditions (20). In patients with ST-segment elevation myocardial infarction, intracoronary or intravenous administration of nicorandil during primary PCI improved myocardial microcirculation and reduced infarct size (21–23). In the placebo-controlled Impact Of Nicorandil in Angina (IONA) trial (24), compared with placebo, nicorandil given in addition to standard anti-anginal treatment significantly decreased the composite endpoint (coronary heart disease death, non-fatal myocardial infarction, or unplanned hospital admission for cardiac chest pain) by 17% in patients with stable angina. In the Japanese Coronary Artery Disease (JCAD) study (25), nicorandil improved all-cause mortality by 35% in patients with CAD. An RCT by Jiang et al. (26) showed that nicorandil therapy reduced ischemic episodes on 24-h Holter monitoring. However, RCTs evaluating the effect of nicorandil in patients with CTO are lacking. Our study used 3D-STE to detect myocardial ischemia, and we found that in patients with coronary CTO, nicorandil therapy for 3 months was associated with a significant improvement in GAS.

Two-dimensional echocardiography is commonly used to detect regional wall-motion abnormalities (RWMA) and decreased LVEF in CAD patients. However, the interpretation of RWMA and the measurement of LVEF are strongly dependent on the skills of the operator. Moreover, changes in RWMA and LVEF may not be present even in patients with CTO. STE enables the objective assessment of global and regional LV myocardial deformation. Myocardial ischemia could lead to changes in LV myocardial deformation that may not be detectable with RWMA and LVEF (27). Recently, 3D-STE has emerged and can provide a comprehensive assessment of all LV segments. Longitudinal, circumferential, and radial strains, which reflect myocardial deformation in the three directions, can be calculated simultaneously using 3D-STE (12, 13). 3D-STE can also provide area strain values, which is a novel strain parameter. Area strain reflects the decrease in endocardial surface area during LV contraction and is related to longitudinal and circumferential myocardial shortening. Studies have shown that GAS has a better diagnostic value for severe coronary stenosis than that provided by other strain parameters. In the study by Li et al., (12), the area under the curve for GLS, GRS, GCS, and GAS was 0.896, 0.866, 0.797, and 0.909, respectively, for the identification of severe stenosis in suspected patients without LV-RWMA. In a study by Dogdus et al. (13), GAS was best correlated with Gensini score, and a GAS value of > −21% had 97.2% sensitivity and 88.1% specificity to detect critical CAD. In our study, the absolute value of GAS and GLS were positively correlated with SAQ scores, but GCS and GRS were not significantly correlated with SAQ scores, indicating that both GAS and GLS are sensitive for detecting myocardial ischemia. However, only GAS was significantly improved by nicorandil treatment. This could be because GAS reflects both longitudinal and circumferential components of myocardial deformation, and is more sensitive for detecting myocardial ischemia than the other strain parameters.

The 19-item SAQ has been used extensively in clinical studies and in clinical practice to assess angina-related health status. SAQ scores are independently associated with the risks of subsequent death, hospitalization, myocardial infarction, and increased health care costs. Furthermore, SAQ has been recommended as a standard outcome measure for patients with CAD by the International Consortium for Health Outcomes Measurement (28). In our study, angina stability and treatment satisfaction improved significantly, and angina frequency decreased significantly, with nicorandil treatment. However, the scores for physical limitation and quality of life were not significantly different between the two groups. This might be because physical limitation and quality of life were also influenced by several factors other than myocardial ischemia (29, 30).

### Limitations

This was a single-center open-label study with small sample size. Thus, the risk of bias must be acknowledged. The follow-up time was short, and the long-term effect of nicorandil requires further investigation. Additionally, patients with heart failure (New York Heart Association class III or IV) were excluded, and most patients included in this study had preserved LVEF. The protective effect of nicorandil could not be extended to all CTO patients. Furthermore, we did not assess myocardial ischemia by stress tests in patients with CTO because we aimed to determine the effect of nicorandil on the improvement of LV myocardial strain in patients with CTO.

## Conclusion

Nicorandil treatment as an adjunct to standard therapy can improve LV myocardial strain and angina symptoms in patients with CTO. Therefore, nicorandil is beneficial for CTO patients.

## Data Availability Statement

The original contributions presented in the study are included in the article/supplementary material, further inquiries can be directed to the corresponding author.

## Ethics Statement

The studies involving human participants were reviewed and approved by the Ethics Review Board of Peking University Third Hospital. The patients/participants provided their written informed consent to participate in this study.

## Author Contributions

SC: data collection and writing the article. CM: patients' following. XF: doing echocardiography. MC: designing the study. All authors contributed to the article and approved the submitted version.

## Funding

This work was supported by the National Natural Sciences Foundation of China (82070272, to MC) and Natural Science Foundation of Beijing Municipality (7202225, to MC).

## Conflict of Interest

The authors declare that the research was conducted in the absence of any commercial or financial relationships that could be construed as a potential conflict of interest.

## Publisher's Note

All claims expressed in this article are solely those of the authors and do not necessarily represent those of their affiliated organizations, or those of the publisher, the editors and the reviewers. Any product that may be evaluated in this article, or claim that may be made by its manufacturer, is not guaranteed or endorsed by the publisher.
